# Minimally Invasive Vaginal Natural Orifice Transluminal Endoscopic Surgery Technique for Successful Polypropylene Mesh Removal in Pelvic Organ Prolapse: A Case Report

**DOI:** 10.7759/cureus.55610

**Published:** 2024-03-05

**Authors:** Nobuo Okui, Machiko Okui

**Affiliations:** 1 Urology, Yokosuka Urogynecology and Urology Clinic, Yokosuka, JPN; 2 Urogynecology, Yokosuka Urogynecology and Urology Clinic, Yokosuka, JPN

**Keywords:** transvaginal mesh, mesh removal, vnotes, vaginal natural orifice transluminal endoscopic surgery, polypropylene mesh, pelvic organ prolapse

## Abstract

In the treatment of pelvic organ prolapse, the insertion of polypropylene mesh is often necessary but can lead to subsequent complications, such as a high incidence of pain and infections, necessitating mesh removal. However, the removal of polypropylene mesh can be challenging due to the risks of postoperative complications and technical difficulties. The key to effective healing often lies in the complete removal of the mesh, but this process is associated with complications, including severe pain and potential foreign body reactions. These challenges underscore the need for less invasive and more precise removal techniques.

In our clinical practice, traditional approaches, such as vaginal and open abdominal surgeries, have often been hindered by limited visibility and accessibility at the mesh fixation sites. To address these issues, our team has pioneered the development of vaginal natural orifice transluminal endoscopic surgery (vNOTES) for mesh removal. This innovative and minimally invasive technique, performed through the vaginal route, holds particular promise for repairs within the pelvic cavity. vNOTES not only enhances surgical visibility but also reduces the invasiveness of the procedure.

In this case report, we present an 85-year-old female patient, who underwent transvaginal mesh (TVM) insertion at the age of 68 years. The patient developed pain in the left buttock, left lower back, and vulvar region, necessitating the removal of TVM. The vNOTES approach significantly reduced postoperative pain and complications, enabling efficient and safe removal of the polypropylene mesh. Moreover, the pathological examination of the polypropylene mesh, which was causing hip and buttock pain, revealed the presence of poor granulation tissue, indicative of a specific pathological tissue pattern. To the best of our knowledge, this is the first detailed account of the successful application of vNOTES in mesh removal.

## Introduction

Pelvic organ prolapse poses a significant health burden and often requires surgical intervention with polypropylene mesh insertion [1]. However, the insertion of polypropylene mesh frequently leads to complications such as pain and infection. The removal of polypropylene mesh, a treatment for these complications, presents considerable challenges due to the risks of postoperative complications and technical difficulties [2-5]. Effective healing hinges on complete mesh removal, yet this process is fraught with complications, including severe pain and potential foreign body reactions [6,7]. These issues underscore the need for a less invasive and more precise removal technique. In routine clinical practice, we have observed that traditional approaches, such as vaginal and open abdominal surgeries, are often hindered by limited visibility and accessibility issues at mesh fixation sites.

To address these challenges, our team pioneered the development of vaginal natural orifice transluminal endoscopic surgery (vNOTES) for mesh removal [8]. This innovative, minimally invasive technique is performed through the vagina and holds particular promise for repairs within the pelvic cavity [9]. vNOTES not only enhances surgical visibility but also reduces the invasiveness of the procedure [10]. We present a case where the vNOTES approach significantly reduced postoperative pain and complications, enabling the efficient and safe removal of polypropylene mesh. To the best of our knowledge, this case report is the first to provide a detailed account of the successful application of vNOTES for mesh removal [2-7].

## Case presentation

Patient information

**Figure 1:**
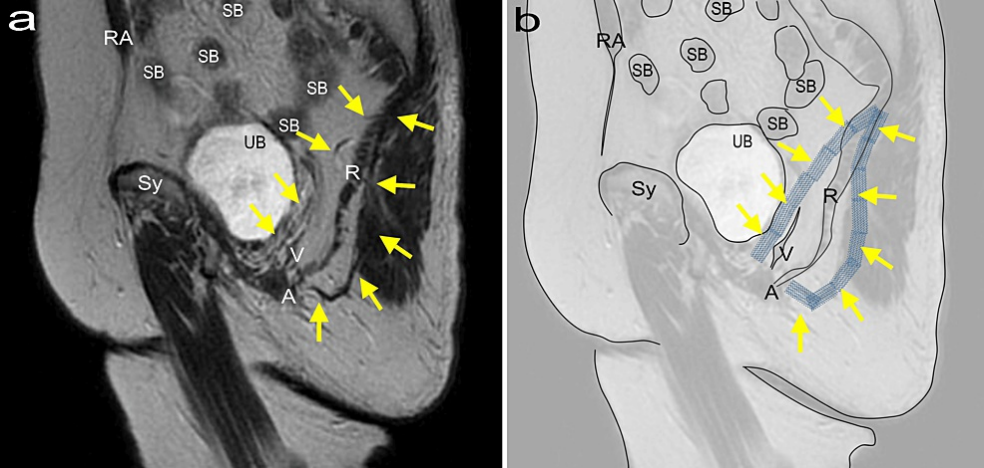
The path and anatomical diagram of polypropylene mesh (a) 1.5T MRI T2-weighted sagittal view, TR 4500 ms, flip angle 140 degrees, TE 100 ms, slice thickness 3 mm, interval 3.5 mm, FOV 32 x 36 cm, matrix 320 x 320 (Signa Creator, GE Healthcare, Chicago, Illinois). (b) Illustration of the image in Panel (a) with emphasis on mesh placement. The yellow arrows indicate the polypropylene mesh (illustrated in Figure 1b to highlight the path of the mesh). RA: Rectus abdominis; SB: Small bowel; R: Rectum; V: Vagina; A: Anus; UB: Urinary bladder; Sy: Symphysis pubis; FOV: Field-of-view; TR: Repetition time; TE: Time to echo. Source: The illustration in Figure 1b is an original work by the author, Nobuo Okui.

The patient is an 85-year-old female. In 2006, the patient underwent repair surgery for pelvic organ prolapse (POP) using polypropylene mesh and hysterectomy, following a diagnosis 20 years before bladder and uterine prolapse at stage 4 severity (POP stage IV) [11]. The discomfort caused by POP disappeared, but the patient experienced left hip pain immediately after surgery. In 2012, she developed severe left hip and buttock pain and was diagnosed with an overactive bladder (OAB). The patient was administered propiverine hydrochloride (20 mg). Regarding pain management, the patient was administered 25-75 mg of diclofenac sodium or 60-120 mg of loxoprofen daily. Pregabalin at a dose of 150 mg was administered. However, these results were not consistently valid. Her OAB symptom score (OABSS) was 7 points [12]. In 2020, her OAB symptoms worsened, her OABSS score increased to 12 points, and she started taking fesoterodine fumarate 8 mg. She complained of abdominal discomfort, urinary leakage, and pain in the external genital area, which affected her daily life. During the pelvic examination, the patient experienced strong pain when the mesh was touched. Imaging (Figure 1) confirmed the presence of mesh, and it was found that the pain along the mesh from the vaginal anterior wall to the left hip matched the pain during palpation and in the lumbar buttock area. Figure 1 (Panel a) shows an MRI image with T2-weighting, and Figure 1 (Panel b) is an illustration highlighting the inserted mesh. An arm-shaped mesh, inserted from 1.5 cm below the anus and 1.5 cm laterally, folded around the sacrococcygeal ligament and extended into the vagina was identified and located between the bladder and vagina.

In the preoperative examination in 2023, the VAS scores were as follows: left hip 10, left waist 10, external genital pain 8, abdominal discomfort 6, and overactive bladder symptom score (OABSS) 12. She also underwent a one-hour pad test, which resulted in 45 g of leakage.

Previous surgical approach

**Figure 2:**
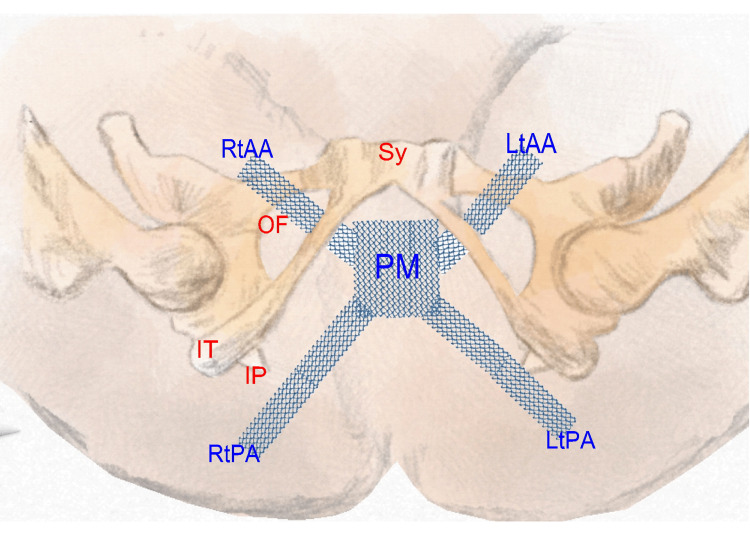
Overview of the predicted polypropylene mesh placement from surgical records PM: Polypropylene mesh; RtAA: Right anterior arm; LtAA: Left anterior arm; RtPA: Right posterior arm; LtPA: Left posterior arm; Sy: Symphysis pubis, IT: Ischial tuberosity; IP: Ischial spine; OF: Obturator foramen. Source: This illustration is an original work by the author, Nobuo Okui.

Figure 2 shows the size and shape of the mesh that was assumed based on surgical records at the time. In 2006, surgery was performed under general anesthesia, including a simple vaginal hysterectomy. During surgery, the left and right uterosacral, round, and sacrospinous ligaments were cut. A polypropylene mesh (GYNEMESH® PS PROLENE®, Johnson & Johnson, Ohio, USA) with four arms was used, with a central part measuring approximately 4 × 3 cm, which was inserted in the bladder-vagina space. Two arms were passed through the pelvic cavity from the skin to the pelvic cavity using the trans obturator (TOT) needle, and the two arms were pulled out from the pelvic cavity to the closure membrane [11]. The posterior intravaginal slingplasty (IVS) was inserted from the left and right sides of the anus, bypassing the sacrococcygeal ligament and passing through the surrounding tissue to reach the vagina, where the remaining two arms were pulled out [13]. This process is referred to as transvaginal mesh (TVM) [14].

Current surgical approach

**Figure 3:**
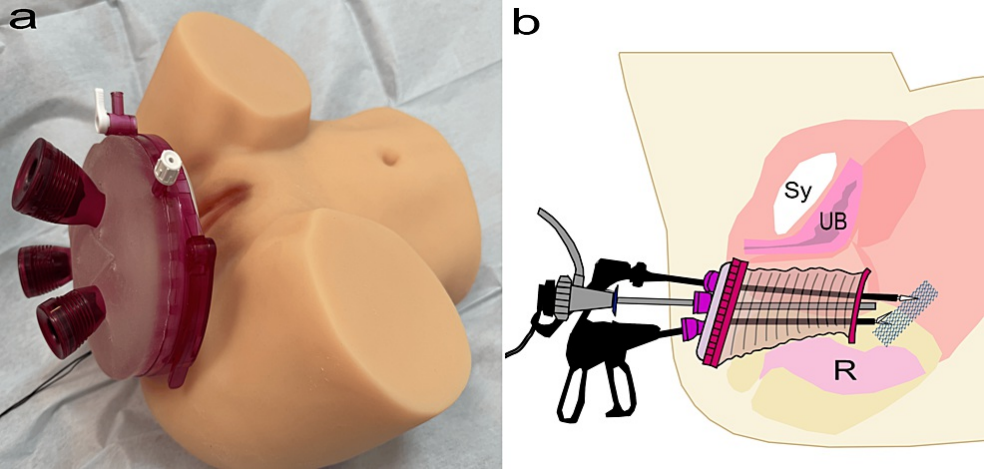
The vNOTES model and illustration (a) A model with the GelPOINT V-Path Transvaginal Access Platform (Applied Medical, Rancho Santa Margarita, California) fixed in place. (b) An illustration depicting the use of the GelPOINT V-Path Transvaginal Access Platform for laparoscopic mesh extraction. Sy: Symphysis pubis; UB: Urinary bladder; R: Rectum; vNOTES: Vaginal natural orifice transluminal endoscopic surgery. Source: Figure 3b is an original work by the author, Nobuo Okui.

Complete mesh removal was planned for this patient. Detaching the insertion site by posterior IVS would require a blinded procedure as it would be difficult to visualize with a vaginal approach. Moreover, inserting a laparoscope from the umbilicus would provide a challenging visual field. Therefore, a polypropylene mesh removal surgery using vNOTES was performed. Figure 3 shows vNOTES. Figure 3a shows the GelPOINT V-Path Transvaginal Access Platform (Applied Medical, Rancho Santa Margarita, California) inserted into the vagina to perform vNOTES, while Figure 3b illustrates the concept of transvaginal mesh access and mesh removal using endoscopy.

General anesthesia was administered, and the patient was placed in the supine position. Antiseptic drapes were applied to both the perineal and abdominal areas to allow for a possible switch to laparotomy or laparoscopy. A urinary catheter was inserted at the start of the procedure, and 2 g cefazolin was administered intravenously as antibiotic prophylaxis.

The first stage of the surgery followed the standard vaginal mesh removal procedure, with a midline incision made in the central part of the vaginal anterior wall to selectively separate the mesh from the tissue and preserve the blood vessels and fascia. The mesh arms inserted into the closed cavity through the vaginal anterior wall were removed, guided by thinning of the vaginal wall along the arms. Partial detachment of the mesh arms facilitated easy removal.

**Figure 4:**
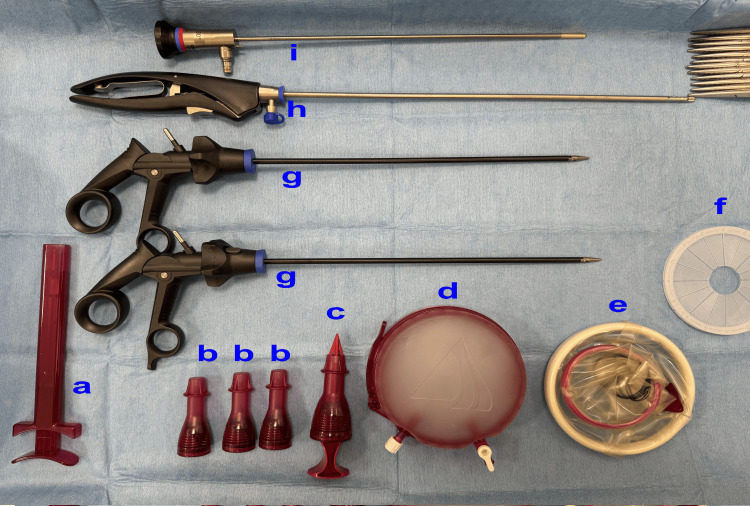
vNOTES model (a) Introducer, (b) sleeve, (c) obturator, (d) gel seal cap, (e) Alexis wound retractor, (f) instrument shield, (g) laparoscopic grasping forceps, (h) laparoscopic scissors, and (i) laparoscopic needle holder (a-f: Applied Medical, Rancho Santa Margarita, California; g-i: Olympus Corporation, Tokyo, Japan). vNOTES: Vaginal natural orifice transluminal endoscopic surgery.

The second stage involved making a 360-degree incision at the vaginal blind end (formerly the site of the uterus), followed by arterial hemostasis. Figure 4 shows the Access Platform GelPoint™ device (Applied Medical, Rancho Santa Margarita, California), endoscopic carbon dioxide insufflation device OLYMPUS UCR, and HICURA-type grasping forceps (Olympus Corporation, Tokyo, Japan). A GelPoint™ device was inserted, and a CO_2_ gas pressure of 8 mmHg was used. A standard 10 mm, 0° rigid laparoscope was inserted through the camera trocar at the 6 o'clock position, with standard laparoscopic instruments inserted through the other two trocars. The laparoscopy equipment included the VISERA ELITE II high-intensity light source unit (OLYMPUS CLV-S190), endoscopic carbon dioxide insufflation device (OLYMPUS UCR), and HICURA-type grasping forceps.

**Figure 5:**
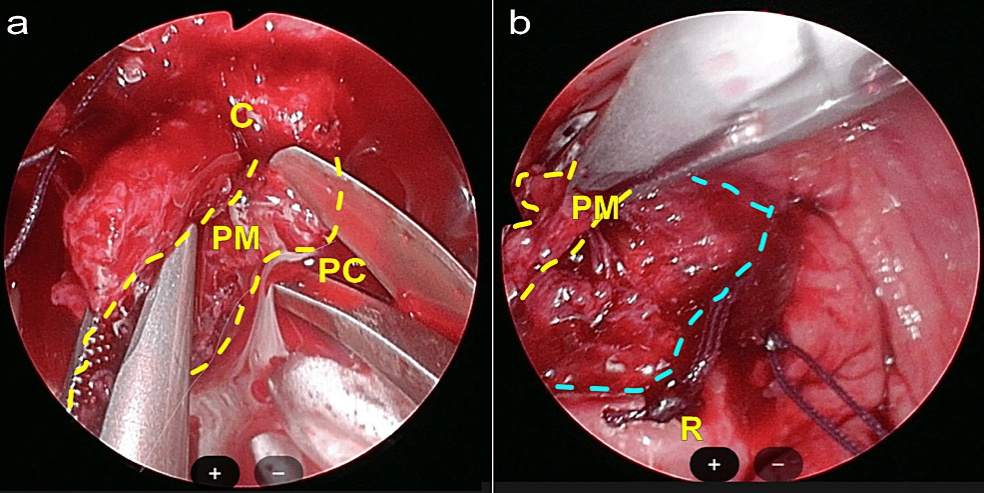
Laparoscopic surgery photos (a) A view showing the area around the border of the coccygeal muscle and pubococcygeus muscle after adhesions have been cleared, with polypropylene mesh inserted, displaying fragile and easily bleeding scar tissue. (b) An image taken by making an incision in the anal direction from Figure 4a, identifying the polypropylene mesh within the adhesion tissue. The yellow-dashed line indicates the overview of the polypropylene mesh, and the blue-dashed line indicates the incision to release the mesh within the uterine removal site. PM: Polypropylene mesh; C: Coccygeal muscle; PC: Pubococcygeus muscle; R: Rectum.

After the laparoscope was set up, the mesh arms on both sides were located. The mesh was embedded in the tissue that was prone to bleeding. Figure 5 depicts the view during vNOTES. When an incision was made in the posterior pelvic wall to inspect the arms connected to the bladder-vaginal mesh endoscopically, the tissue was fragile, necessitating careful dissection. Minimal incisions were made to detach the mesh from the sacrum (Figure 5, Panel a) and posterior pelvic wall (Figure 5, Panel b), allowing extraction by pulling on the mesh. In particular, areas where the patient experienced pain had poor granulation tissue around the mesh. The GelPoint device was removed through an incision at the vaginal base.

The third stage of surgery involved passing two angled sutures through the edges of the sacrospinous ligament, which was confirmed during the second stage, and suturing the anterior and posterior vaginal flaps. Additionally, vaginal wall sutures were used to prevent the recurrence of POP. This third process is referred to as native tissue repair (NTR) [15].

The surgery lasted 120 min, and the estimated blood loss was 92 ml. The patient was discharged on the following day.

Surgical outcome

The surgery proceeded as planned, and nearly all the polypropylene mesh was successfully removed using vNOTES endoscopic techniques. Improved visualization during surgery allowed for precise mesh removal.

Recovery and progress

**Figure 6:**
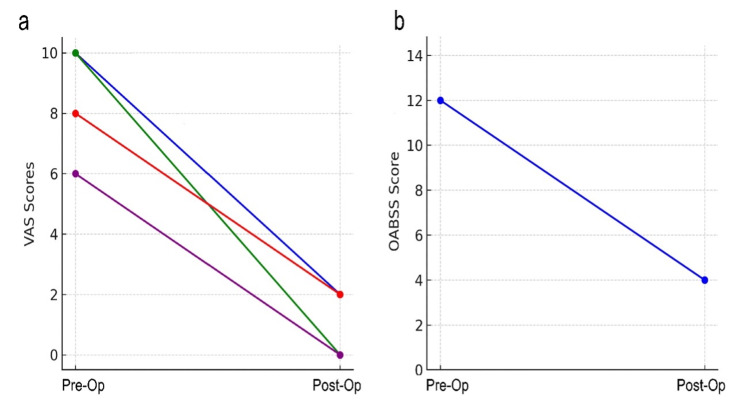
Changes in pain and OAB before and after the surgery (a) Changes on VAS. The blue line indicates left hip pain; the green circle indicates left flank pain; the red circle indicates perineal pain; and the purple circle indicates lower abdominal pain. (b) Changes in OABSS. The horizontal axis represents before and after surgery. VAS: Visual analog scale, OABSS: Overactive bladder symptom score.

The patient resumed physical activity shortly after surgery, and pain relief was rapid. The patient was discharged after a one-day hospital stay. Figure 5 shows that symptoms of urinary leakage and abdominal discomfort improved dramatically, allowing the patient to return to daily life. At the three-month postoperative follow-up, the VAS scores were as follows: left hip 2, left waist 0, external genital pain 2, abdominal discomfort 0, and OABSS 4 (Figure 6, Panels a and b). The one-hour pad test resulted in 5 g of leakage.

Pathological study

**Figure 7:**
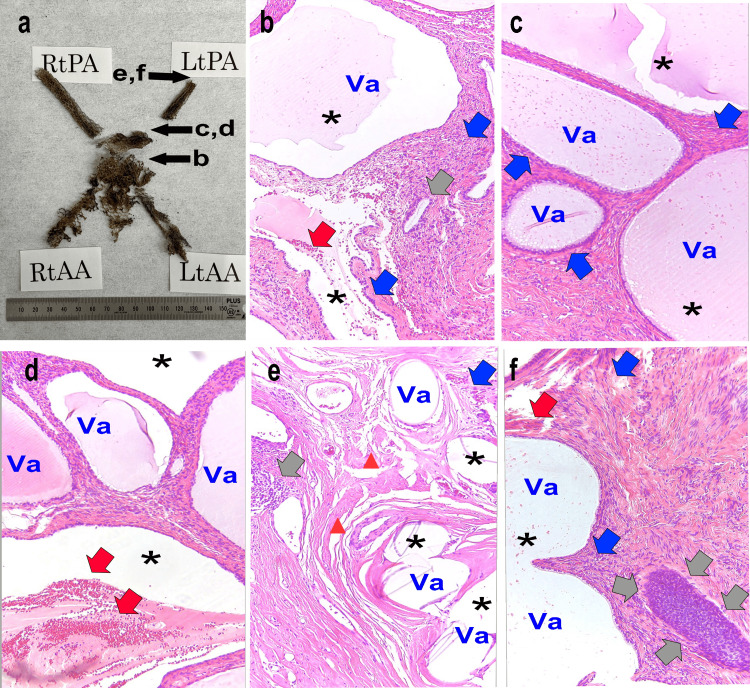
Extracted mesh and pathological tissues (a) Photograph showing the extracted mesh arranged in a dried, inserted, and fixed format. The black arrows and labels b-f indicate areas where part of the mesh is missing, corresponding to the parts excised in the respective pathology photographs (panels b-f). (b) Histological image of the mesh inserted between the bladder and vagina. (c and d) Histological images near the boundary between the coccygeal muscle and the pubococcygeal muscle. This area is a cause of back pain. (e and f) The region causing left buttock pain is excised by incising toward the anal direction for comparison with Figure 6b. The gray arrows indicate the incomplete granulation due to a foreign body; the red arrows indicate hemorrhage; the blue arrows indicate foreign body giant cells; the red closed triangle indicates the rupture of muscle fibers; and * indicates the evidence of tears in the blank shot state due to the polypropylene mesh fibers breaking down from age-related degradation. The images in panels b-f were stained with hematoxylin and eosin (H&E) and captured at an optical magnification of 40x. Va: Vacuoles formed due to the mesh peeling off during the preparation of the pathological specimen; RtPA: Right posterior arm; LtPA: Left posterior arm; RtAA: Right anterior arm; LtAA: Left anterior arm.

Figure 7 shows the macro- and micro-aspects of the pathology in the mesh and surrounding tissues. Figure 7 (Panel a) depicts the overall view of the extracted mesh, and it was possible to remove a mesh of nearly the same size as the initial prediction (Figure 2). In Figure 7 (Panel b), the mesh that was strongly adhered between the bladder and vagina was noticeable for its bleeding (red arrows) and the formation of a significant void (Va) due to the mesh. Foreign body giant cells were present throughout (blue arrows). In Figure 7 (Panels c and d), the tissue from the site of left hip pain is shown. During specimen preparation, an area containing the artificial mesh became detached, forming a gap (Va). Numerous empty spaces (Va) were observed around multinucleated giant cells (blue arrows). In Figure 7 (Panels e and f), the mesh within the muscles of the hip, where the detachment was challenging during surgery, is shown. Cyst formation (Va) and foreign body granulation (gray arrows) are observed. Inflammatory changes indicated the suppression of normal cell proliferation and the proliferation of aberrant granulation tissue. In all fields of view, there were areas where the surrounding tissue was crushed or the formation of voids was enlarged owing to the breakdown of polypropylene fibers (*). Additionally, this breakdown resulted in the rupture of muscle fibers (red triangles).

## Discussion

The successful application of vNOTES for the removal of polypropylene mesh in our patient indicates a significant advancement in the surgical management of complications following POP repair. Traditional methods, such as vaginal and open abdominal surgeries, are limited by issues such as restricted visibility and accessibility, particularly at mesh fixation sites. In contrast, vNOTES offers enhanced visualization and minimally invasive access, effectively addressing several of these challenges.

The high anatomical success rate of using a polypropylene mesh with the TVM technique has been well documented in previous studies [11]. In cases of both POP and OAB, anatomical correction achieved through the TVM technique has been reported to lead to an improvement in OABSS [11]. However, long-term follow-up has shown decreasing success rates and significant complications, including pain and infection, necessitating mesh removal [4]. In our case, there was not only pain but also an increase in OABSS. Wang et al. investigated the effects of polypropylene mesh degradation on tissue inflammatory responses, while Vancaillie et al. defined pain after POP repair surgery with mesh as post-surgical neuropathy, emphasizing the need for treatment [2,3]. However, it may take time for a surgeon to notice the patient's pain. Todd et al. focused on the measurement methods for pain associated with mesh and pointed out a lack of consistency in pain management and assessment [16]. Alibrahim et al.'s case report took 13 years to resolve the pain caused by the mesh [5]. Our case indicates the need for improved surgical techniques for mesh removal, and it shows that resolution can take a long time.

The case of our patient aligns with other instances showcasing vNOTES's capability for meticulous dissection and thorough mesh removal, thus signifying its role in enhancing postoperative outcomes. A notable concern in surgeries involving mesh and hysterectomy for POP treatment, such as in our patient, is the potential for spontaneous recurrence after TVM surgery. This complexity is compounded by the risk of foreign body reactions and inflammation around the mesh and adjacent tissues, which are common pathological findings in such cases [17]. Although direct studies on vNOTES in this context are limited, the minimally invasive nature of the procedure suggests the possibility of reduced tissue trauma and inflammatory response, which are critical factors for postoperative recovery and long-term tissue integration.

In particular, the development of voids around the polypropylene mesh and the formation of suboptimal granulation tissue were observed in our patient, which correlated with complaints of lower back and buttock pain. These pathological changes are significant as they often indicate inadequate tissue healing and integration of the mesh. While specific literature on vNOTES’s impact on such tissue responses is scarce, the precision and minimal invasiveness of vNOTES could theoretically minimize extensive tissue disruption, potentially leading to better tissue healing and less postoperative pain. In studies focusing on the removal of polypropylene mesh for mid-urethral sling procedures in stress urinary incontinence, similar findings were observed, with void formation around the polypropylene and the presence of foreign body giant cells and abscesses, as in our case report [7]. Prior research has also shown a correlation between the location of pain and specific pathological features [7]. Further research is needed to determine the effects of vNOTES on tissue healing and granulation tissue quality in POP surgeries.

The rapid postoperative recovery and a significant reduction in pain and symptoms further validated the effectiveness of this approach. Lauterbach et al. reinforced this point, showing that vNOTES hysterectomy significantly improves surgical performance compared to traditional vaginal surgery. These include shortened operative time, reduced blood loss, minimal post-surgical pain, quick recovery, and shorter hospitalization, all of which contribute to a substantial improvement in the quality of life post-surgery [18]. Previous studies have also reported rapid pain improvement following mesh removal [7]. Considering our patient's case, in conjunction with these findings, it is plausible that the mesh contributed to anatomical abnormalities. Additionally, as shown in previous studies, NTR surgeries without mesh have higher subjective satisfaction and no risk of exposure to prosthetic material compared to TVM [19]. Ultimately, as recommended in prior research, the complete removal of all mesh and conversion to NTR could have contributed to pain improvement.

Our experience suggests that vNOTES is a viable alternative to traditional mesh removal methods, offering a less invasive approach with fewer complications. A study by Chaccour et al. supports this, showing that vNOTES hysterectomy is non-inferior to laparoscopic hysterectomy, with the benefits of shorter operative and recovery times, reduced postoperative pain, and fewer complications. However, it is also important to note that vNOTES was found to be more expensive, which could affect its accessibility and adoption [20]. This finding highlights the need for skilled surgeons trained in endoscopic techniques to ensure the safety and effectiveness of this approach. Future studies with larger patient cohorts and long-term follow-up are essential to establish the generalizability and long-term efficacy of vNOTES for mesh removal after POP repair surgeries.

## Conclusions

The vNOTES technique for the removal of the polypropylene mesh represents a substantial advancement in the surgical management of postoperative complications associated with POP repair. As demonstrated in this case, vNOTES offers a less invasive alternative with enhanced visibility, which facilitates precise mesh removal and potentially leads to improved patient outcomes, including reduced pain and quicker return to daily activities. The successful application of this technique in our 85-year-old patient underscores its potential as a new standard for addressing intricate challenges associated with mesh-related complications.

Pathologically, the mesh-induced buttock and back pain in our patient was significant, with aberrant cells and poor granulation tissue, suggesting a complex foreign body reaction far from normal tissue patterns. The delicate and meticulous removal required in such cases highlights the importance of vNOTES as an increasingly necessary surgical option. The vNOTES approach allows for careful dissection and complete extraction of mesh, addressing the pathological issues at hand and paving the way for more effective and patient-centered surgical interventions in the future.
